# Factorial Structure and Validity of Depression (PHQ-9) and Anxiety (GAD-7) Scales after Traumatic Brain Injury

**DOI:** 10.3390/jcm9030873

**Published:** 2020-03-23

**Authors:** Ali Teymoori, Anastasia Gorbunova, Fardzadeh E. Haghish, Ruben Real, Marina Zeldovich, Yi-Jhen Wu, Suzanne Polinder, Thomas Asendorf, David Menon, CENTER-TBI Investigators and Participants, Nicole v. Steinbüchel

**Affiliations:** 1 The Department of Social Psychology, Helmut Schmidt University, 22043 Hamburg, Germany; 2 The Institute of Medical Psychology and Medical Sociology, Georg August University, 37073 Göttingen, Germany; Anastasia.Gorbunova@med.uni-goettingen.de (A.G.); haghish.ebad-fardzadeh@med.uni-goettingen.de (F.E.H.); r.real@web.de (R.R.); marina.zeldovich@med.uni-goettingen.de (M.Z.); yi-jhen.wu@med.uni-goettingen.de (Y.-J.W.); thomas.asendorf@med.uni-goettingen.de (T.A.); nvsteinbuechel@med.uni-goettingen.de (N.v.S.); 3 Department of Public Health, Erasmus University Medical Center, 3000 Rotterdam, The Netherlands; s.polinder@erasmusmc.nl; 4 Institute of Medical Statistics, Medical Center, Georg August University, 37073 Göttingen, Germany; 5 Division of Anaesthesia, University of Cambridge/Addenbrooke’s Hospital, Box 157, Cambridge CB2 0QQ, UK; dkm13@wbic.cam.ac.uk

**Keywords:** depression, anxiety, bifactorial model, validity, comorbidity

## Abstract

Background: The dimensionality of depression and anxiety instruments have recently been a source of controversy. Objectives and Design: In a European-wide sample of patients after Traumatic Brain Injury (TBI), we aim to examine the factorial structure, validity, and association of the Patient Health Questionnaire for depression (PHQ-9) and the Generalized Anxiety Disorder (GAD-7) instruments. This study is based on longitudinal observational data. We conducted analyses of factorial structure and discriminant validity of outcomes six-months after TBI. We also examined the prevalence, co-occurrence, and changes of scores on the PHQ-9 and GAD-7 at 3-, 6-, and 12-month post-TBI assessments. Participants: At six-months post-TBI assessment, 2137 (738 (34.5%) women) participants completed the PHQ-9 and GAD-7 questionnaires. For the longitudinal analysis, we had 1922 participants (672 (35.0%) women). Results: The results of exploratory factor analysis suggested a general latent construct underlying both PHQ-9 and GAD-7 measures. Confirmatory factor analyses showed a slight improvement in the fit indices for the bifactorial model. The Omega hierarchical test clearly differentiated two subfactors of PHQ-9 and GAD-7 items over and above the underlying general factor; however, most of the variance (85.0%) was explained by the general factor and the explained variance of the subfactors was small. The PHQ-9 and GAD-7 performed similarly in detecting post-traumatic stress disorder (PTSD). As defined by conventional cut-offs, depression and anxiety have different prevalence rates in the sample. The scales also differed in their relationships with the short form of health survey (SF-36v2) subscales. The longitudinal analysis showed high stability of depression and anxiety symptoms: 49–67% of the post-TBI patients with comorbid depression and anxiety reported the persistence of the symptoms over time. Discussion: The factorial structure analysis favors a general latent construct underlying both depression and anxiety scales among patients after TBI. We discuss the implications our findings and future research directions.

## 1. Introduction

Depression and anxiety are among the most commonly experienced mental health disorders in both the general population and different medical conditions [1,2]. However, depression and anxiety are often under-recognized in medical settings [3], and this can complicate the patients’ recovery [4].

Among individuals with a history of Traumatic Brain Injury (TBI), depression and anxiety are the most common mental health problems [5,6]. TBI is characterized by alterations of brain function, including loss of consciousness, impaired memory, neurological deficit (e.g., loss of balance), and alteration of mental state at the time of injury [7]. TBI severity varies from mild to moderate and severe, and over 90% of the TBI cases are mild TBIs [7]. The prevalence of TBI is high, with 50 million annual worldwide occurrences: Nearly half of the world’s population will suffer from some form of TBI at least once over their lifetime [7].

When remaining untreated, depression and anxiety not only impede the patient’s recovery from TBI, but also leave a lasting impairment in their post-TBI life, such as poorer social functioning and more post-concussion symptoms [8,9,10]. The prevalence rate of depression among patients after TBI is estimated to be between 15% and 27% [8,11] and for anxiety between 23% and 29% [5,10,12].

There is a growing body of literature reporting the coexistence and comorbidity of depression and anxiety [13,14,15]. The comorbidity of depression and anxiety has also been found in TBI [9]. As a result, there is a growing call to investigate depression and anxiety together and further examine their relationship and comorbidity not only cross-sectionally but also over time [5].

Furthermore, conceptually, depression and anxiety are distinct constructs, although they are part of the same category of mood disorders. For instance, anxiety was predominantly conceptualized as an unpleasant feeling and worrying reaction to a perceived future threat and depression as a diminished activity and loss of pleasure and interest [16].

Empirically, the factorial structure analysis of depression and anxiety has resulted in contradictory evidence not only in the composite scale of depression and anxiety (such as Depression Anxiety Stress Scales—21 (DASS-21): [17]; Mood and Anxiety Symptom Questionnaire (MASQ): [18]; Hospital Anxiety and Depression Scale (HADS): [19]), but also in the scales that aim to measure the two constructs of depression and anxiety separately. In the composite measurement of depression and anxiety, using HADS for instance, previous research proposed various hierarchical structure of this scale including a one-factorial [20], two-factorial [21,22], three-factorial [23], and a bifactorial model [24,25]. With the use of a bifactorial model, which depicts a general psychological distress factor encompassing all items and two orthogonal grouping subfactors of depression and anxiety, most variance was explained by the general factor and the subfactors’ contribution was negligible [24,25]. Consequently, a systematic review of studies on the latent structure of HADS cast doubt on the ability of this measure to reliably differentiate depression and anxiety and proposed a general latent construct of psychological distress underlying the HADS measure [26].

Regarding the measurements that aim to screen patients with symptoms of depression and anxiety separately, the widely used instruments are the nine-item Patient Health Questionnaire (PHQ-9; [27,28]) and the seven-item scale for Generalised Anxiety Disorder (GAD-7; [29]). Both PHQ-9 and GAD-7 items are based on the DSM-IV criteria for depression and anxiety [27,29] and proved to be valid in a variety of different medical settings [see 10] including for patients after TBI, e.g., [5,30].

Dimensionality has not been thoroughly scrutinized on a composite measure of PHQ-9 and GAD-7. In most studies, they were considered to be independent and unidimensional instruments (for a review, see [10]), with few studies showing an extra somatic subdimension besides the cognitive affective subdimension in both PHQ-9 and GAD-7 (e.g., [31]). Recently, Kroenke et al. [32] combined the PHQ-9 and GAD-7 items and with the use of bifactorial modeling proposed a composite measure of depression and anxiety called the Patient Health Questionnaire Anxiety and Depression Scale (PHQ-ADS). According to the bifactorial modelling terminology, PHQ-ADS is the general factor and the two PHQ-9 and GAD-7 scales are the subfactors. Despite the negligible contribution from the subfactors to the explanation of scale variance, Kroenke et al. [32] stated that PHQ-ADS does not override the value of individual PHQ-9 and GAD-7 subfactors. However, they did not provide the evidence showing the independent effect of PHQ-9 and GAD-7 over and above PHQ-ADS. In this study, we examine the factorial structure of a composite measure PHQ-ADS in TBI and evaluate the subfactors PHQ-9 and GAD-7′s discriminant, construct, and predictive validity. We also look at the cross-sectional and longitudinal comorbidity of depression and anxiety.

In sum, the aims of the present study are (1) to investigate the relation between scores of PHQ-9 and GAD-7 instruments, (2) the factorial structure of the composite measure of PHQ-ADS, and (3) the discriminant, construct, and predictive validity of the subfactors of PHQ-9 and GAD-7. We examine the study objectives in a European sample of patients after TBI.

## 2. Methods

### 2.1. Participants

This study uses data from the Collaborative European NeuroTrauma Effectiveness Research in TBI (CENTER-TBI (Core 2.0)) project [33]. CENTER-TBI is a longitudinal observational study involving 59 different medical and research centers in 18 countries. The CENTER-TBI study included 4509 patients with a clinical diagnosis of TBI [34]. The inclusion criteria included being recruited within 24 h after TBI, clinical indication for a CT-scan, lack of severe neurological disorders before the injury, and informed consent [33]. We selected those who were 16 years of age or above (*n* = 4360) and survived the 6-months post TBI (*n* = 3886). For the factorial structure analysis, we used the 6-month post-TBI data, in which 2137 participants (738 (34.5%) women, 1399 (65.5%) men) completed the psychological outcome measures of PHQ-9 and GAD-7. These participants were recruited from three strata including patients primarily admitted to the intensive care unit (ICU, 885 (41.4%) at the time of enrollment), patients admitted to hospital ward (admission stratum, 805 (37.7%)), and patients evaluated in the emergency room and discharged (ER, 447 (20.9%)). The cause of injury was mainly due to road accidents (40.71% of incidence) and incidental fall (43.61%) and few patients reported their injury to be due to other causes such as assault (3.93%) and suicide attempt (0.89%).

For the predictive validity and sequential comorbidity of depression and anxiety, we analyzed 1922 patients that had at least two out of the three time-point assessments (3, 6, and 12 months post-TBI: 672 (35.0%) women, 1250 (65.0%) men), from which 794 (41.3%) patients were recruited from the ICU, 761 (39.6%) from the admission stratum, and 376 (19.1%) from the ER.

### 2.2. Ethical Approval

The CENTER-TBI study has been conducted in conformance with all relevant local national ethical guideline and regulatory requirements for recruiting human subjects, as well as with relevant data protection, privacy regulations, and informed consent. The study obtained ethical clearance from both the EU and the relevant institutions across all countries that were involved in the project. Informed consent was also obtained from all participants according to national and local procedures (for a list of sites, ethical committees, and ethical approval details, see https://www.center-tbi.eu/project/ethical-approval).

### 2.3. Instruments

Demographic information was collected at the time of enrollment into the study and included participants’ sex, age, and educational background.

The Glasgow Coma Scale (GCS) assesses coma and impaired consciousness after TBI with scores that were obtained at several time points within 24 h post-injury such as pre-hospital, first arrival at hospital, and post-stabilization [35]. Following the IMPACT methodology [36], GCS scores were based on the post-stabilization period, and when the score was not available at the post-stabilization stage, the previous non-missing scores were used. The GCS categorizes injury into severe (3–8), moderate (9–12), and mild (13–15).

The Glasgow Outcome Scale, Extended (GOSE) was assessed at 6-months post-TBI to assess functional disability and recovery after TBI [37]. The GOSE classifies functional outcomes into eight categories: Dead (1), vegetative state (2), lower severe disability (3), upper severe disability (4), lower moderate disability (5), upper moderate disability (6), lower good recovery (7), and upper good recovery (8). In our study, we had patients with a GOSE score of 3 or above.

The PHQ-9 assesses the frequency and severity of symptoms of depression using nine 4-point Likert-scaled items ranging from 0 (not at all) to 3 (nearly every day) [27] (Kroenke & Spitzer, 2002). A total score ranging from 0 to 27 is obtained by summing across all items, with ordinary mean substitution for missing items if no more than one third (no more than three items) are missing. The total score can be categorized at a cutoff of 10 to differentiate between minimal/mild versus moderate/severe depression [15,38]. However, a systematic review indicated that a cutoff of 8 might increase sensitivity to depression [39]. We used both cut-off points. 

The GAD-7 measures the frequency and severity of generalized anxiety disorder symptoms [29], using seven 4-point Likert-scale items with a response format ranging from 0 (not at all) to 3 (nearly every day). A total score ranging from 0 to 21 was obtained by summing all items with ordinary mean substitution for missing items if less than one third (less than two items) were missing. The total score can be categorized at a cutoff of 10 [15] or 8 to optimize the test’s sensitivity and specificity for identifying other anxiety disorders [40].

The PHQ-ADS combines the PHQ-9 and GAD-7 scales as composite measure of mental distress [32].

The Short-Form-36v2 (SF-36v2; [41]) is a generic measure of physical and mental outcomes with eight dimensions: Physical Functioning (PF, 10 items), Role Limitation due to Physical Health (RP, 4 items), Role Limitation due to Emotional Health (RE, 3 items), Social Functioning (SF, 2 items), Body Pain (BP, 2 items), Vitality (VT, 4 items), Mental Health (MH, 5 items), General Health (GH, 5 items). SF-36 is commonly used to measure TBI people’s health-related quality of life [42]. Higher scores indicate better health-related quality of life.

The PTSD Checklist for DSM-5 (PCL-5; [43]) is a self-report instrument to assess symptoms of post-traumatic stress disorder (PTSD) across four symptoms clusters defined in the DSM-5 (reexperiencing, avoidance, negative alterations in cognition and mood, and hyperarousal). Intensity is assessed using 20 five-point Likert scaled items ranging from 0 (not at all) to 4 (extremely). A total sum score exceeding a cutoff of 33 indicates a likely PTSD [43,44].

We used the translated versions of the PHQ-9 and GAD-7 questionnaires that were available in the relevant languages (see https://www.phqscreeners.com/select-screener/36) and were shown to be valid and invariant across different sex, patient strata, and languages [45]. Following a procedure recommended by Acquadro, Conway, Giroudet, and Mear [46], GOSE and PCL-5 were translated, back-translated, panel-reviewed, and finally underwent a cognitive debriefing with several patients and healthy participants. The final versions were revised based on the cognitive debriefing results to ensure the conceptual and linguistic equivalence of the scales with the original English language.

### 2.4. Statistical Analysis

We examined three different aspects of the PHQ-9 and GAD-7 tests: (a) Reliability, (b) factorial validity using exploratory and Confirmatory Factor Analyses (CFA) and Omega hierarchical test, and (c) discriminant, construct, and predictive validity.

Reliability: Reliability was first analyzed using Cronbach’s alpha and Lambda 2 of Guttmann’s coefficient (λ_2_) separately for PHQ-9 and GAD-7 as well as for the composite measure of depression and anxiety (PHQ-ADS). The item level descriptive statistics of the PHQ-ADS showed many skewed-distributed items (Appendix A), creating a floor effect and violating the normality assumption. Moreover, using the original response format, the initial CFA analysis shows dissatisfactory model fit indices even with the use of ordinal estimator, the Weighted Least Square Mean and Variance adjusted (WLSMV). Consequently, to examine the factorial structure, we dichotomized the items (see the note in Appendix A), with two values: 0 (no depression/no anxiety) and 1 (some degree of depression/anxiety, collapsing the original scores from 1 to 3 into 1).

Factorial validity: Principal Axis Factoring (PAF) was used to determine the number of factors for PHQ-ADS in a parallel analysis of the scree plots with the Kaiser–Guttman criterion. To confirm the factorial structure of PHQ-ADS items, we used CFA with WLSMV estimator and theta parameterization for ordinal variables. We compared models with a one-, two-, and bifactorial structure. Finally, to examine the factorial structure reliability, we used McDonald’s Omega Hierarchical test (OmegaH).

The OmegaH test evaluates the bifactorial structure of the scale, examining how the factorial structure of the PHQ-ADS can be accounted for by: (a) A single general factor (g), representing the shared explained variance of all items, and (b) a set of group factors in which additional variance over and above the general factor can be explained by a subset of similar items [47,48]. As a result, the OmegaH test examines the extent to which the test reflects a common trait and is a good test for dimensionality of the latent construct. We used several different parameters from Omega test including Omega general for the general factor and subfactors, OmegaH, Explained Common Variance (ECV), and Percentage of Uncontaminated Correlation (PUC) [49,50]. Omega general assesses the unit weighted variance of the general factor and the subfactors, whereas OmegaH estimates the proportion of variance that can be attributed to the general factor, treating the variance due to the subfactors as measurement error [48]. The ECV provides an assessment of the relative strength of the subfactors, examining the variance explained by general factor divided by variance explained by both general factor and subfactors [48]. In conjunction with ECV, PUC evaluates the percentage of unique correlation estimating the general factor, providing additional information whether there is a bias in considering the scale as unidimensional. Higher PUC values indicate lower variability in ECV coefficient. Sufficient evidence of unidimensionality in the Omega hierarchical test of the bifactorial model depends of the ECV coefficient (≥0.70), PUC (≥0.70), and OmegaH coefficient (≥0.70) [48].

We used the omega function with oblique rotation method, tetrachoric correlation matrix given the ordinal nature of the rescaled responses, and the Schmid–Leiman transformation for a bifactorial model [51]. Following suggestions from Rodrigues et al., [50] and Bonifay et al., [49], we conducted various validity tests to further examine whether the subfactors of PHQ-9 and GAD-7 have distinct explanatory contribution.

Discriminant validity: For discriminant validity, we compared the performance of PHQ-9 and GAD-7 in detecting an anxiety related disorder, PTSD. We hypothesized that GAD-7 would have a better odds ratio in classifying patients with PTSD correctly than would PHQ-9 [29,40]. For this purpose, we applied logistic regression and cross tabulation for an odds ratio of correct classification. 

Construct and predictive validity: For construct validity, we examined the relationship of PHQ-9 and GAD-7 with the SF-36v2. Using the longitudinal data, we examined whether anxiety precedes depression, as assumed in previous literature (e.g., [52]). For this purpose, we analyzed the 3-, 6-, and 12-month data. To do so, we divided the participants into two groups based on their GAD-7 score at 3 or 6 months. Then we applied the McNemar’s test for the intra-individual changes of depression symptom over time in each of the low and high anxiety group [53]. 

Sequential comorbidity. Finally, we examined the sequential comorbidity of depression and anxiety using the McNemar–Bowker with a pairwise post-hoc analysis, which is an extension of McNemar test for more than two groups. With the McNemar–Bowker test, we examined whether the contingency table is symmetric such that the probability of cell (i, j) is equal to the probability of change in the cell (j, i), and a significant result would indicate a change from i to j. We relied on Cohen’s [54] criteria to assess the odds ratio (*OR*: Small effect <1.86, medium for 1.86–3.00, and large for ≥3.00) and Cohen’s *g* (small effect size for <0.15, medium for 0.15–0.25, large for ≥0.25).

All analyses were conducted using R version 3.5.3, and the packages “lavaan” [55], and “psych” [51]. We exported the data from the Neurobot platform of the CENTER-TBI (https://center-tbi.incf.org/) and used the “CENTER Core 2.0” dataset.

## 3. Results

### 3.1. Respondents Characteristics

The basic demographic information and medical characterization of the study participants (at six-months post-TBI assessment) for the overall sample, as well as for each sex separately, can be seen in Table 1. The participants had a wide age range (*M* = 49.19, *SD* = 19.30, *Mdn* = 51, range = 16–95), relatively high education background (1585 (83.1%) completed high school education or more), and diverse linguistic backgrounds (see Table 1). The GOSE score showed that more than half of the participants (62.8%) had good recovery, a quarter had moderate disability (25.7%), and 10.3% had severe disability at six months after TBI.

### 3.2. Descriptive of Variables and Their Reliability

Table 2 shows the descriptive statistics, reliability coefficients, and the intercorrelation of the study variables. The mean scores for depression (PHQ-9, *M* = 5.07, *SD* = 5.35, range = 0–27) and anxiety symptoms (GAD-7, *M* = 3.63, *SD* = 4.54, range = 0–21) were low. Cronbach’s alpha and Guttman’s lambda 2 of PHQ-9, GAD-7, and PHQ-ADS showed very good internal consistency. The correlation between PHQ-9 and GAD-7 was high (*r* = 0.80).

### 3.3. Factorial Structure of PHQ-ADS

As explained in statistical analysis section (also see the descriptive statistics in Table 2 and Appendix A), we dichotomized responses to 0 (no depression/anxiety) and 1 (some depression/anxiety). In the parallel analysis (see Appendix B), only one factor seemed to fit the data based on the eigenvalue of more than one (eigen values: 1st factor = 9.81, 2nd = 0.47, 3rd = 0.32), a sharp break in the scree plot between the first and the second factor, and the explained variance (explained variance = 0.61).

We compared model fit indices of one-, two-, and bifactorial models estimated within CFA framework. As can be seen in Table 3, all fit indices had a slight improvement in the bifactorial model. The chi-square was significant for all models, as is the case with the large sample size; however, the ratio of χ^2^-to-df in the two-factorial and bifactorial models was below 3, a threshold that Kline [56] put for a good fitting model. In the two-factorial model, the correlation between PHQ-9 and GAD-7 was high (*r* = 0.91, *p* < 0.001), implying that one dimension might be able to measure both factors [57]. The bifactorial model seems to be the best fitting model based on the slightly better fit indices.

### 3.4. Factorial Structure Reliability

The Omega coefficients for the general factor (ω = 0.97) and the subscales of depression (ω = 0.92) and anxiety (ω = 0.94) were excellent. The OmegaH coefficient for a bifactorial model of PHQ-ADS was 0.87, meaning that 87% of the variance of unit-weighted total scores can be attributed to individual differences in the general factor (see Appendix C). As a result, most of the reliable variances can be explained by the general factor and only 10% (ω (0.97)−ω_h_ (0.87)) of the variance in individuals scores caused by distinct subfactors of PHQ-9 and GAD-7. The ECV coefficient was 0.85, indicating that the 85% of the common variance was captured by the general factor and the remaining 15% is spread across subfactors. The general factor explains most of the variance using OmegaH and ECV coefficients and the subfactors contribution is low in accounting for the individuals’ difference in depression and anxiety. The PUC coefficient was rather low (*PUC* = 0.53), implying that perhaps there would be a bias in considering the test as unidimensional based on the result of ECV.

The percent of common variance due to the general factor was also calculated for each item (Item Explained Common Variance: I-ECV) and higher scores in I-ECV (≥0.85) indicates the contribution to the general factor and little contribution to the subfactors which was the case for majority of the items in this study (Appendix D).

Overall, the omega hierarchical test suggests that GAD-7 and PHQ-9 fit a unidimensional construct better than the bifactorial model. Further validity tests can examine whether, based on the result of PUC coefficient, there is a bias in considering the test unidimensional.

### 3.5. Discriminant Validity

We first categorized the patients based on the cut-off point of 33 in PCL5 into two categories with 0 (no/low PTSD) and 1 (moderate/severe PTSD) values. Logistic regression showed that both PHQ-9 (*B* = 0.20, *z* = 8.54, *p* < 0.001, *OR* = 1.22 (95% *CI*: 1.16–1.27)) and GAD-7 (*B* = 0.20, *z* = 7.85, *p* < 0.001, *OR* = 1.22 (95% *CI*: 1.16–1.28)) significantly associated with PCL-5. We also categorized the PHQ-9 and GAD-7 to test which scale would better detect participants with moderate or severe PTSD cases. With the strict cut-off point of 10 for PHQ-9 and GAD-7, the odd ratios (OR) of correctly classifying the PTSD cases were 28.73 (95% *CI*: 19.85–41.59) and 28.11 (95% *CI*: 19.80–39.90), respectively. With the lenient cut-off point of 8 for PHQ-9 and GAD-7, the *OR* of correct classification of PTSD cases were 30.8 (95% *CI*: 19.92–47.62) and 29.83 (95% *CI*: 20.80–42.79), respectively.

We categorized the PHQ-ADS into two low and moderate/severe groups based on the cut-off point of 20 [32] and obtained a higher OR of correct classification of PTSD cases (*OR* = 41.56, 95% *CI*: 28.76–60.06).

### 3.6. Prevalence, Comorbidity, and Construct Validity

With the use of a stricter cut-off point of 10, the proportion of moderate to severe depression and anxiety symptoms in our study were 18% and 11%, respectively. With the cut-off point of 8, the prevalence of moderate and moderately severe depression and anxiety were 25% and 16% respectively. Using the cut-off point of 8, the majority of participants had neither depression nor anxiety (1526 participants, 72%), and among the rest, 288 (14%) participants had comorbid depression and anxiety, 245 (12%) had only depression, and 53 (3%) had only anxiety. A total of 54% (288 out of 535) of the moderately/severely depressed patients had comorbid anxiety, and 86% (288 out of 341) of moderately/severely anxious patients, had comorbid depression.

**Figure 1:**
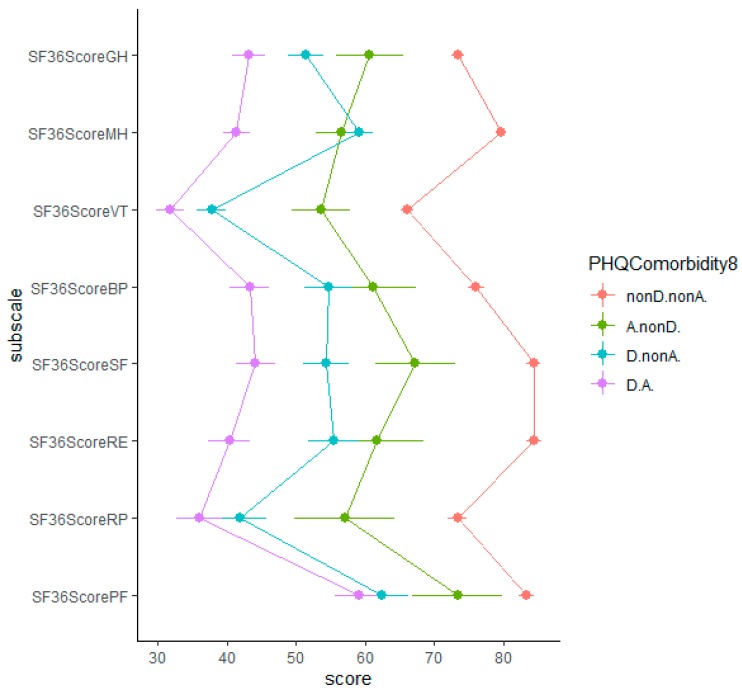
The association of depression and anxiety with physical and mental component scores from the SF-36v2. Note: PF = Physical Functioning; RP = Role Limitation due to Physical Health; RE = Role Limitation due to Emotional Health; SF = Social Functioning; BP = Body Pain; VT = Vitality; MH = Mental Health; GH = General Health; nonD.nonA. = neither depression nor anxiety; A.nonD. = only anxiety; D.nonA. = only depression; D.A. = comorbid depression and anxiety; with the use of cut-off point of 8 for PHQ-9 and GAD-7; the error bar represents the 95% confidence interval.

We examined the association of the PHQ-9 and GAD-7 with the SF-36. The correlation of PHQ-9 and GAD-7 with SF-36 dimensions was significant (Table 2). PHQ-9 had a stronger relationship with SF-36 dimensions than did GAD-7. Individuals with comorbid depression and anxiety had the lowest scores on various dimensions of the physical and psychological outcomes and individuals with neither depression nor anxiety had the highest physical and psychological outcomes score on all dimensions (Figure 1, for the same analysis with the cut-off point 10 for PHQ-9 and GAD-7 see Appendix E).

### 3.7. Predictive Validity and Changes of Depression and Anxiety Over Time

Using the cut-off point of 8 for PHQ-9 and GAD-7, there seems to be a significant intra-individual changes of depression symptoms from three months to six and 12 months, depending on whether individuals have anxiety in the preceding assessment (Table 4). However, the change of depression symptoms was not in the direction that was expected as more participants moved from high depression category to the no/low depression category than the other way around. Individuals’ depression between six and 12 months did not change as a result of high anxiety. We also conducted a logistic regression to predict anxiety and depression at six months based on the preceding depression and anxiety symptoms. In comparison to the participants with no depression and no anxiety, the participants with only depression symptoms at three months had a higher likelihood of showing anxiety symptoms at six months (*B* = 7.67, *z* = 8.54, *p* < 0.001, *OR* = 5.69 (95% *CI*: 3.64–8.88)) than did the participants with only anxiety at three months have in showing depression at six months (*B* = 0.97, *z* = 2.38, *p* < 0.001, *OR* = 2.64 (95% *CI*: 1.11–5.61), see Appendix F). Thus, based on the result of McNemar test and logistic regression, our results did not support the hypothesis that anxiety precedes depression. 

**Figure 2:**
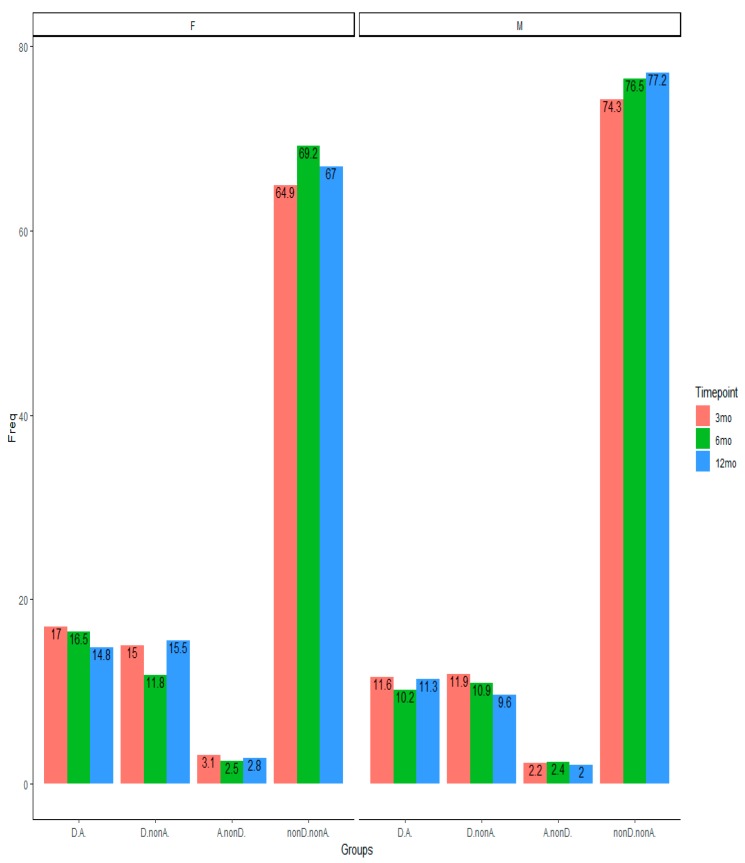
The prevalence (%) of depression and anxiety categories at three time points of 3, 6, and 12 months. Note: F = female; M = male. nonD.nonA. = neither depression nor anxiety; A.nonD. = only anxiety; D.nonA. = only depression; D.A. = comorbid depression and anxiety.

In the longitudinal assessment, women had a higher rate of depression and anxiety than men at all time points (Appendix G). We analyzed the prevalence of the comorbid depression and anxiety over time (Figure 2) and tested whether individuals changed their group category over time (see Appendix H for the contingency table). Looking at the distribution of participants at the whole table, 75.9% of participants from three to six months, 75.3% from three to 12 months, and 79.7% from six to 12 months stayed in the same depression and anxiety category. The preceding symptoms had an impact on later rate of depression and anxiety. Between 88.4% and 90.0% of those with no depression and anxiety at three-month assessment had no depression and anxiety at six and 12 months post-TBI injury and between 49.6% and 66.9% of comorbid depression and anxiety still had comorbid depression and anxiety up to 12 months post-TBI (Appendix H). The McNemar–Bowker Test showed a negligible change in the probability of off-diagonal change in the contingency table from three to six months (OR = 1.43, *p* = 0.558, Cohen’s *g* = 0.089), from three to 12 months (OR = 1.30, *p* = 0.565, Cohen’s *g* = 0.065), and from six to 12 months (OR = 1.30, *p* = 0.566, Cohen’s *g* = 0.066). Thus, there were no significant differences in probability of change from depression to anxiety, and vice versa (see Appendix H for contingency Table A4).

## 4. Discussion

In this study, we examined the factorial structure of a composite measure of depression and anxiety and evaluated the validity of the subfactors. The results of exploratory and confirmatory factor analyses, as well as the Omega hierarchical test, suggested a unidimensional construct such that both instruments were part of a common general factor, as the subscales of PHQ-9 and GAD-7 separately had negligible independent effects to explain the variance of the construct. This is partly understandable as there are a few similar items in both the subscales, such as being restless and having trouble relaxing/sleeping. In addition, both depression and anxiety symptoms are part of the same mood disorder category, share a common domain of negative affect, and share a cognitive process with negative bias in information processing.

The subfactors of PHQ-9 and GAD-7 performed very similarly in detecting an anxiety-related disorder, PTSD. Examining the construct validity, both PHQ-9 and GAD-7 subfactors were negatively related to various domains of physical and psychological outcomes, with PHQ-9 having noticeably stronger negative relationship with SF-36 subscales than GAD-7 (Table 2, Figure 1). The negative relationship with SF-36 subscales was much higher for patients with comorbid depression and anxiety. In addition, the result of our longitudinal analysis did not support the hypothesis that anxiety precedes depression, as proposed in the literature [52]. Perhaps it is the nature of the traumatic experiences such as TBI that provides a common cause for the experience of both depression and anxiety symptoms. On the other hand, it is the social avoidance aspect of the anxiety that is deemed to be a risk factor for the onset of depression [59,60]. Unfortunately, we did not have a measure for social anxiety in our study. Besides that, the TBI incidence, the subsequent medical care and potential social support, as well as the post-TBI recovery process might complicate the relevance of social avoidance aspect of anxiety for the onset of depression symptoms.

The depression and anxiety symptoms seemed to be stable over time and the small developmental changes pattern from general anxiety to depression symptoms and vice versa were rather symmetrical up to one year after TBI. Similarly, in a birth cohort that was followed for 32 years, Moffitt et al. [61] found that depression also precedes general anxiety symptoms as often as the reverse pattern. Thus, the developmental changes from one to another requires us to take both pathways into account and study it with other types of anxiety symptoms as well (for a review, see [59]).

Our findings concern patients after TBI, and this should be borne in mind when seeking explanations of why depression and anxiety are so closely related. It would be interesting to find out what shared cognitive processes, or shared biological pathways, contribute to the strong relationship between depression and anxiety. However, it is not clear if the unidimensionality is because of the specific traumatic experience in TBI that provides a shared common cause for both depression and anxiety. Comparative works with depression and anxiety after other types of traumatic events, e.g., other stressful life events, and comparison with control group can shed more light on this.

Furthermore, it is not clear whether the lack of unique effect of depression and anxiety subfactors is due to the self-report nature of PHQ-9 and GAD-7 measures. Is the difference between these constructs more experiential that might be better captured through other research method? Using mixed methods such as the diagnostic interviews and phenomenological analysis in addition to the self-report measure can better clarify this point. For instance, with using HADS and structural interviews, Whelan-Goodinson, Ponsford, and Schönberger [62] found that HADS is a reliable measure of a general psychological distress construct for patients after TBI and cannot differentiate depressed and anxious cases identified through structural interviews.

To test the predictive validity, we had only data from three, six, and 12 months after TBI, and it is not clear if anxiety does not precede depression or if this is not a sufficient time period for anxious patients to develop depression. As already explained, we included only the general anxiety disorder and there might be another sub-type of anxiety, such as social anxiety, that might be a risk factor for the onset of depression. A more comprehensive longitudinal test requires data for a longer period and information on different sub-types of anxiety symptoms (e.g., social anxiety, phobias), familial and contextual factors, as well as the pre-TBI depression and anxiety symptoms. Finally, a more detailed investigation between PHQ-ADS and more related constructs such as bipolar disorder and suicidality are required. For instance, one of the items in PHQ-9 has been contested to measure suicidality rather than depression [38], although the criteria remained in the recent edition of DSM-IV.

Furthermore, we have not investigated the association between depression and other physical, social, and clinical functioning of the TBI patients given the characteristics of our sample. Teymoori et al. [45] found that the functional recovery is a much more important predictor of depression and anxiety severity than the baseline TBI severity, such that individuals with severe and moderate disability six months after the TBI had higher depression and anxiety symptoms in comparison to individuals who had a good recovery after the TBI. Noteworthy, TBI severity was not related to depression and anxiety after controlling for functional recovery measure [45]. Further analysis regarding the association of depression and anxiety with other physical, social, and clinical functioning should a task that can be addressed in the future studies.

The debate about whether depression and anxiety scales can actually differentiate the depressed and anxious people is not new (e.g., see [63]). On the other hand, the result of studies on other scales, such as HADs, has already shown such inability to differentiate these two constructs through self-reporting measures. In addition, the high comorbidity and strong correlation between depression and anxiety might indicate a lack of distinctiveness in measuring what they aim to measure (also see [64]). Our study shows that PHQ-ADS as a general unidimensional measure is valid and reliable to use for screening patients for depression and anxiety after TBI and has a good performance in detecting related construct such as PTSD. Notwithstanding, for an accurate clinical diagnosis and treatment purposes, detailed medical and psychiatric/psychological evaluations are required.

## Figures and Tables

**Table 1:** Descriptive characteristics of the sample.

Variable	Overall	Stratified	
*n*	2137	Female (738)	Male (1399)
Countries (*n*, (%))			
The Netherlands	498 (23.3)	199 (40)	299 (60)
Italy	269 (12.6)	84 (31.2)	185 (68.8)
Norway	260 (12.2)	81 (31.2)	179 (68.8)
Spain	254 (11.9)	76 (29.9)	178 (70.1)
The UK	207 (9.7)	70 (33.8)	137 (66.2)
Finland	207(9.7)	83 (40.1)	124 (59.9)
Belgium	154 (7.2)	45 (29.2)	109 (70.8)
Sweden	60 (2.8)	28 (46.7)	32 (53.3)
France	52 (2.4)	9 (17.3)	43 (82.7)
Others	176 (8.2)	63 (35.8)	113 (64.2)
Age groups (*n*, (%))			
(16–24)	325 (15.2)	95 (29.2)	230 (70.8)
(25–34)	262 (12.3)	78 (29.8)	184 (70.2)
(35–44)	274 (12.8)	67 (24.5)	207 (75.5)
(45–54)	357 (16.8)	130 (36.4)	228 (63.9)
(55–64)	397 (18.6)	146 (36.8)	251 (63.2)
(> = 65)	521 (24.4)	222 (42.6)	299 (57.4)
Patient stratum (*n*, (%))			
ER	447 (20.9)	198 (44.3)	249 (55.7)
Admission	805 (37.7)	297 (36.9)	508 (63.1)
ICU	885 (41.4)	243 (27.5)	642 (72.5)
GOSE (*n*, (%))			
Severe dis. (3, 4)	221 (10.3)	81 (36.7)	140 (63.3)
Moderate dis. (5, 6)	549 (25.7)	185 (33.7)	364 (66.3)
Good recovery (7, 8)	1341 (62.8)	459 (40.9)	882 (65.8)
NA	26 (1.2)	13 (50)	13 (50)
GCS (*n*, (%))			
Severe	340 (16.4)	90 (26.5)	250 (73.5)
Moderate	162 (7.8)	55 (34)	107 (66)
Mild	1571 (75.8)	575 (36.6)	996 (63.4)
Education level (*n*, (%))			
None	23 (1.2)	12 (52.2)	11 (47.8)
Currently studying	58 (3.0)	22 (37.9)	36 (62.1)
Primary school	240 (12.6)	86 (35.8)	154 (64.2)
Secondary/high school	651 (34.1)	213 (32.7)	438 (67.3)
Post high school	402 (21.1)	109 (27.1)	293 (72.9)
University/college	533 (27.9)	209 (39.2)	324 (60.8)

ER = Emergency Room; Admission = patients admitted to hospital wards; ICU = Intensive Care Unit, GOSE = The Glasgow Outcome Scale, Extended; Severe dis. = patients with severe disability; Moderate dis. = patients with moderate disability.

**Table 2:** The descriptive statistics, reliability coefficients, and the correlation between variables.

	α	λ2	*M*	*SD*	Min–Max (Q1, Q2, Q3)	GAD-7	PHQ-9	PHQ-ADS
GAD-7	0.91	0.91	3.63	4.54	0–21 (0, 2, 6)	1.00		
PHQ-9	0.87	0.88	5.07	5.35	0–27 (1, 3, 8)	0.80	1.00	
PHQ-ADS	0.94	0.94	8.71	9.40	0–48 (2, 6, 13)	0.94	0.96	1.00
PCL-5	0.93	0.94	12.20	13.61	0–80 (0, 8, 17)	0.76	0.76	0.80
SF-36, PF	0.94	0.94	77.31	26.89	0–100 (65, 90, 100)	−0.32	−0.42	−0.39
SF-36, RP	0.95	0.94	64.18	31.94	0–100 (40, 69, 100)	−0.44	−0.55	−0.53
SF-36, RE	0.94	0.91	74.56	28.33	0–100 (50, 83, 100)	−0.60	−0.65	−0.66
SF-36, SF	0.85	-	75.06	26.49	0–100 (63, 87, 100)	−0.56	−0.67	−0.65
SF-36, BP	0.88	-	68.79	27.00	0–100 (51, 74, 100)	−0.45	−0.52	−0.51
SF-36, VT	0.84	0.83	57.77	21.86	0–100 (44, 62, 75)	−0.58	−0.73	−0.70
SF-36, MH	0.86	0.84	71.49	20.04	0–100 (60, 75, 90)	−0.76	−0.76	−0.80
SF-36, GH	0.76	0.73	66.41	22.28	0–100 (50, 70, 82)	−0.51	−0.59	−0.58

PHQ-ADS: The Patient Health Questionnaire Anxiety and Depression Scale. *M* = mean, *SD* = standard deviation. Q1 = first quartile, 25%; Q2 = second quartile or median, 50%; Q3 = third quartile, 75%. PF = Physical Functioning; RP = Role Limitation due to Physical Health; RE = Role Limitation due to Emotional Health; SF = Social Functioning; BP = Body Pain; VT = Vitality; MH = Mental Health; GH = General Health. Notes: α: Cronbach’s alpha coefficient; λ_2_: Lambda 2 of Guttman coefficient; all correlation coefficients were significant at *p* < 0.001.

**Table 3:** Summary of the Confirmatory Factor Analyses (CFA) for single, two, and bifactorial models of depression and anxiety.

	*χ*^2^ (*df*)	*χ*^2^/*df*	*p*	RMSEA ^a^	^b^ RMSEA 95% CI	^c^ SRMR	^d^ CFI	^e^ TLI	^f^ NFI	^g^ IFI
**One-factorial M.**	477.63 (104)	4.49	<0.001	0.042	0.038–0.045	0.048	0.995	0.994	0.994	0.995
**Two-factorial M.**	308.46 (103)	2.99	<0.001	0.031	0.027–0.035	0.039	0.997	0.997	0.996	0.997
**Bifactorial M.**	189.76 (88)	2.15	<0.001	0.024	0.019–0.028	0.032	0.999	0.998	0.998	0.999

^a^ RMSEA = root mean squared error of approximation; ^b^ RMSEA (95% CI) = 95% confidence interval of root mean squared error of approximation; ^c^ SRMR = standardized root mean square residual; ^d^ CFI = comparative fit index; ^e^ TLI = Tucker–Lewis index; ^f^ NFI = the Normed Fit Index, ^g^ IFI = incremental fit index. Notes: Traditionally, the standard cut-offs for RMSEA (<0.06) and CFI, TLI, NFI, and IFI (>0.95) indices were used to indicate acceptable model fit [58]. However, these cut-offs are not validated for WLSMV estimator and therefore we did not interpret them directly but used them for model comparisons.

**Table 4:** The McNemar test statistics (chi-squared) for intra-individuals changes of depression over time in low and high anxiety groups using the McNemar chi-squared test.

	**Generalized Anxiety Disorder (GAD-7) Categories at 3-Month Based on the Cut-Off Point of 8**										
	**Low Anxiety at 3-Months**						**High Anxiety at 3-Months**				
Matched variable		**Depression at 6 Mo**						**Depression at 6 Mo**			
		0, *n* (%)	1, *n* (%)	Total	Mc (df), *p* *			0, *n* (%)	1, *n* (%)	Total	Mc (df), *p*
Depression at 3 mo	0	1056 (91.6)	**97 (8.4)**	1153	0.18 (1), *p* = 0.61	**Depression at 3 mo**	0	33 (80.5)	**8 (19.5)**	41	42.61 (1), *p* < 0.001
	1	**103 (48.6)**	109 (51.4)	212			1	**63 (28.0)**	162 (72.0)	225	
	Total	1159 (84.9)	206 (15.1)				Total	96 (36.1)	170 (63.9)		
	**Generalized Anxiety Disorder (GAD-7) categories at 3-month based on the cut-off point of 8**										
	**Low anxiety at 3-Months**						**High anxiety at 3-Months**				
		**Depression at 12 Mo**						**Depression at 12 Mo**			
		0, *n* (%)	1, *n* (%)	Total	Mc (df), *p*			0, *n* (%)	1, *n* (%)	Total	Mc.(df), *p*
Depression at 3 mo	0	713 (89.6)	**83 (10.4)**	796	0.94 (1), *p* = 0.33	**Depression at 3 mo**	0	17 (70.8)	**7 (29.2)**	24	23.17 (1), *p* < 0.001
	1	**71 (47.7)**	78 (52.3)	149			1	**40 (31.5)**	87 (68.5)	127	
	Total	784 (83.0)	161 (17.0)				Total	57 (37.7)	94 (62.3)		
	**Generalized Anxiety Disorder (GAD-7) categories at 6-month based on the cut-off point of 8**										
	**Low anxiety at 6-Months**						**High anxiety at 6-Months**				
		**Depression at 12 Mo**						**Depression at 12 Mo**			
		0, *n* (%)	1, *n* (%)	Total	Mc (df), *p*			0, *n* (%)	1, *n* (%)	Total	Mc (df), *p*
Depression at 6 mo	0	818 (90.5)	**86 (9.5)**	904	8.38 (1), *p* < 0.01	**Depression at 6 mo**	0	20 (62.5)	**12 (37.5)**	32	0.86 (1), *p* = 0.353
	1	**52 (39.1)**	81 (60.9)	133			1	**17 (12.8)**	116 (87.2)	133	
	Total	870 (83.9)	167 (16.1)				Total	37 (22.4)	128 (77.6)		

* McNemar’s chi-squared (degree of freedom), *p*-value. Bold: Highlighting the asymmetrical change of depression from earlier to later time point, i.e., individuals that changed their cells over time.

**Figure A1:**
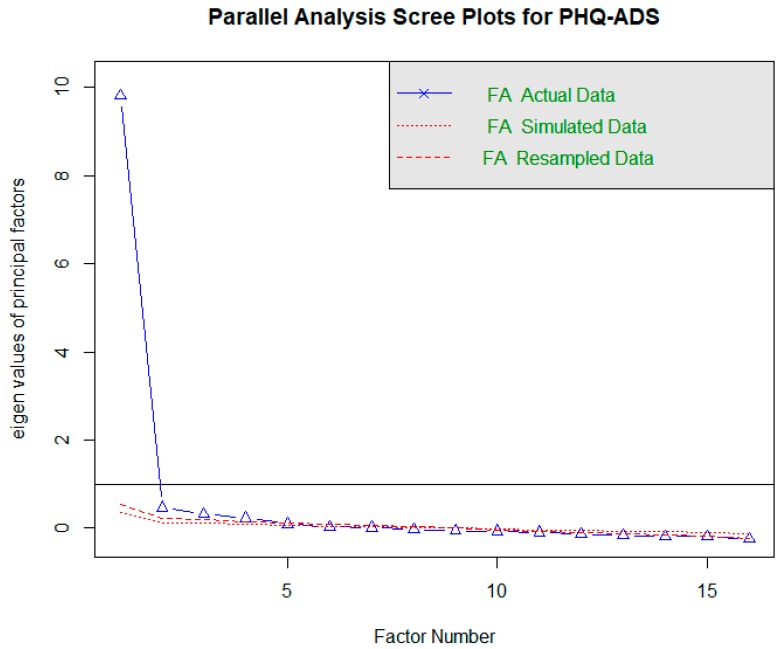
The Scree plot for a composite measure of Patient Health Questionnaire Anxiety and Depression Scale (PHQ-ADS).

**Figure A2:**
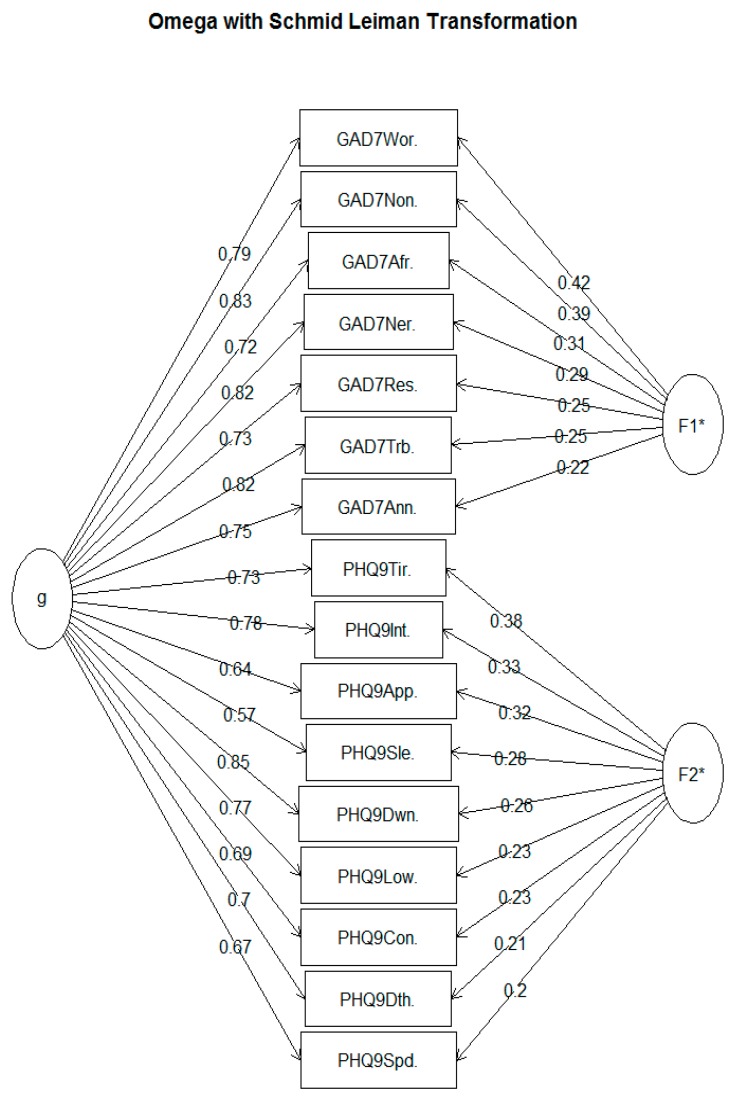
The bifactorial model for Patient Health Questionnaire for depression (PHQ-9) and seven-item scale for Generalised Anxiety Disorder (GAD-7).

**Figure A3:**
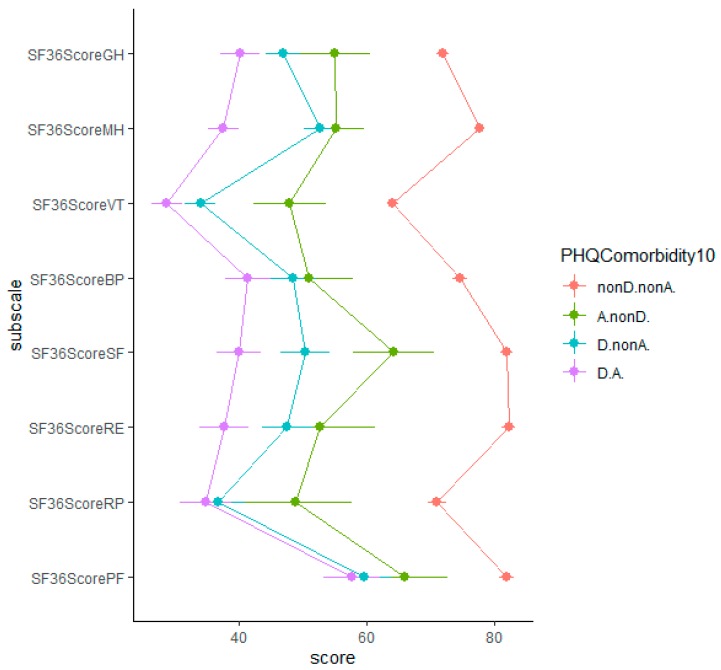
The relative impact of depression and anxiety on the physical and psychological outcomes with the cut-off point of 10 for PHQ-9 and GAD-7.

**Figure A4:**
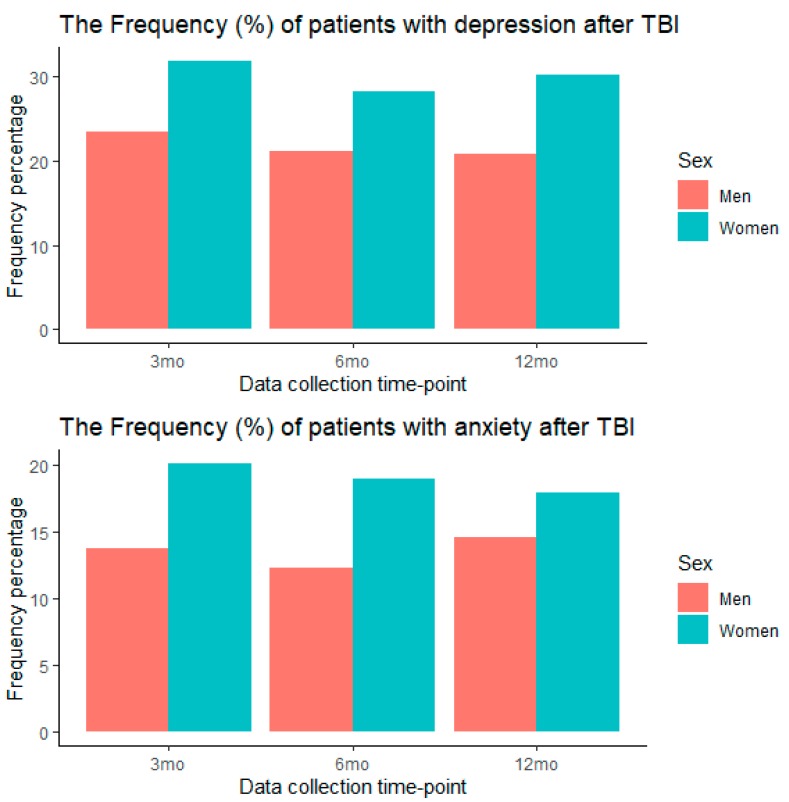
The percentage of sample with depression and anxiety for women and men.

## Supplementary Materials

The following are available online at https://www.mdpi.com/2077-0383/9/3/873/s1, The CENTER-TBI participants and investigators.

## Appendix A

**Table A1:** Descriptive statistics of the items.

	*n*	Mean	S.D.	Min-Max (Q1, Median, Q3)	Skewness	Kurtosis
GAD7 (Nervous)	2122	0.60	0.80	0–3 (0, 0, 1)	1.36	1.42
GAD7 (NonStopWorry)	2120	0.52	0.83	0–3 (0, 0, 1)	1.61	1.81
GAD7 (WorryTooMuch)	2121	0.62	0.86	0–3 (0, 0, 1)	1.34	1.00
GAD7 (TrblRelax)	2121	0.58	0.85	0–3 (0, 0, 1)	1.45	1.32
GAD7 (Restless)	2120	0.36	0.71	0–3 (0, 0, 1)	2.14	4.17
GAD7 (Annoyed)	2122	0.59	0.84	0–3 (0, 0, 1)	1.41	1.25
GAD7 (Afraid)	2112	0.35	0.73	0–3 (0, 0, 0)	2.26	4.61
PHQ9 (IntrstPleasrActScre)	2125	0.60	0.86	0–3 (0, 0, 1)	1.44	1.32
PHQ9 (DwnDeprssnHopelssScre)	2126	0.55	0.80	0–3 (0, 0, 1)	1.47	1.56
PHQ9 (SleepProbScre)	2125	0.86	1.03	0–3 (0, 1, 1)	0.91	−0.42
PHQ9 (TirdLckEnrgyScre)	2126	1.03	0.99	0–3 (0, 1, 2)	0.69	−0.56
PHQ9 (AppteIssueScre)	2123	0.52	0.87	0–3 (0, 0, 1)	1.64	1.71
PHQ9 (LowSlfEstmScre)	2121	0.42	0.77	0–3 (0, 0, 1)	1.93	3.04
PHQ9 (ConcntrtnProbScre)	2124	0.58	0.88	0–3 (0, 0, 1)	1.46	1.17
PHQ9 (SpdMovmntSpchScre)	2124	0.31	0.71	0–3 (0, 0, 1)	2.47	5.53
PHQ9 (DthHrtThghtScre)	2121	0.18	0.53	0–3 (0, 0, 0)	3.53	13.50

Note: As can be seen in the descriptive statistics, most participants answered that they were not at all or just a few days of the week bothered by the symptoms of the depression and anxiety. As a result, most participants selected the lower end of the item responses (0 or 1). For instance, the first quantile, median, and third quantile for the first item of the PHQ-9 was 0, 0, and 1, and for the first item of GAD-7 was also 0, 0, 1, respectively. As another example, in response to a question as to on how many days of the last two weeks individuals were afraid something awful might happen (Item 7 of GAD-7), 1607 participants were not bothered at all (0), 341 for just several days (1), 85 for more than half the days (2), and 79 participants nearly every day (3).

## Appendix D

**Table A2:** Standardized Factor Loadings for the General Factor and the Two Sub-Factors of Depression and Anxiety in Omega Test and CFA.

	Schmid–Leiman						CFA			
Items	Gen.	Anx.	Dep.	Communality	EV	I-ECV	Gen.		Anx.	Dep.
**1. PHQ9Int.**	0.78		0.33	0.72	0.28	0.85	0.83 ***		0.15 **	
**2. PHQ9Dwn.**	0.85		0.26	0.81	0.19	0.89	0.93 ***		−0.02	
**3. PHQ9Sle.**	0.57		0.28	0.41	0.59	0.80	0.59 ***		0.28 ***	
**4. PHQ9Tir.**	0.73		0.38	0.68	0.32	0.78	0.76 ***		0.52 *	
**5. PHQ9App.**	0.64		0.32	0.50	0.50	0.80	0.67 ***		0.19 ***	
**6. PHQ9Low.**	0.77		0.23	0.66	0.34	0.89	0.84 ***		−0.06	
**7. PHQ9Con.**	0.69		0.23	0.54	0.46	0.89	0.73 ***		0.10 *	
**8. PHQ9Spd.**	0.67		0.20	0.50	0.50	0.89	0.72 ***		−0.02	
**9. PHQ9Dth.**	0.70		0.21	0.55	0.45	0.89	0.76 ***		−0.12	
**10. GAD7Ner.**	0.82	0.29		0.76	0.24	0.88	0.84 ***	0.26 ***		
**11. GAD7Non.**	0.83	0.39		0.85	0.15	0.82	0.81 ***	0.45 ***		
**12. GAD7Wor.**	0.79	0.42		0.80	0.20	0.78	0.74 ***	0.57 ***		
**13. GAD7Trb.**	0.82	0.25		0.76	0.24	0.89	0.83 ***	0.26 ***		
**14. GAD7Res.**	0.73	0.25		0.60	0.40	0.88	0.73 ***	0.26 ***		
**15. GAD7Ann.**	0.75	0.22		0.63	0.37	0.90	0.77 ***	0.18 ***		
**16. GAD7Afr.**	0.72	0.31		0.62	0.38	0.84	0.72 ***	0.28 ***		

I-ECV: Item Explained Common Variance; EV: Error Variance Note: Schmid–Leiman Factor loadings greater than 0.2; all factor loadings are statistically significant, *p* < 0.001). For CFA: *** for *p* < 0.001; ** for *p* < 0.01; * for *p* < 0.05.

## Appendix F

**Table A3:** Depression and anxiety groups at three months predicting depression and anxiety (cut-off point of 8) at six months using logistic regression.

	PHQ9 at 6-Months			GAD7 at 6-Months		
3 months	**Beta (ER)**	**Z**	**OR (95%CI)**	**Beta (ER)**	**Z**	**OR (95%CI)**
**A.nonD**	0.97 (0.41)	2.38 ***	2.64 (1.11–5.61)	2.25 (0.37)	6.04 ***	9.53 (4.45–19.46)
**D.nonA**	2.44 (0.17)	14.08 ***	11.52 (8.22–16.23)	1.74 (0.23)	7.67 ***	5.69 (3.64–8.88)
**D.A.**	3.33 (0.18)	18.26 ***	27.99 (19.69–40.29)	3.56(0.20)	17.74 ***	35.21 (23.93–52.64)

* *p* < 0.05; ** *p* < 0.01; *** *p* < 0.001.

## Appendix H

**Table A4:** The cross-table of depression and anxiety categories (cut-off point of 8).

	6 Mo					12 Mo				
3 Mo	nonD.nonA.	A.nonD.	D.nonA.	D.A.	Total	nonD.nonA.	A.nonD.	D.nonA.	D.A.	Total
nonD.nonA.	1035 (90.0)	18 (1.6)	67 (5.8)	30 (2.6)	1150	703 (88.4)	9 (1.1)	46 (5.8)	37 (4.7)	795
A.nonD.	27 (65.9)	6 (14.6)	2 (4.9)	6 (14.6)	41	11 (45.8)	6 (25.0)	1 (4.2)	6 (25.0)	24
D.nonA.	100 (47.2)	3 (1.4)	70 (33.0)	39 (18.4)	212	66 (44.6)	4 (2.7)	51 (35.5)	27 (18.2)	148
D.A.	50 (22.2)	13 (5.8)	39 (17.3)	123 (54.7)	225	32 (25.2)	8 (6.3)	24 (18.9)	63 (49.6)	127
Total	1212 (74.4)	40 (2.5)	178 (10.9)	198 (12.2)	1628	812 (74.2)	27 (2.5)	122 (11.2)	133 (12.2)	1094
6 Mo										
nonD.nonA.	-	-	-	-	-	804 (89.1)	12(1.3)	54 (6.0)	32 (3.5)	902
A.nonD.	-	-	-	-	-	13 (40.6)	7 (21.9)	4 (12.5)	8 (25.0)	32
D.nonA.	-	-	-	-	-	49 (36.8)	3 (2.3)	56 (42.1)	25 (18.8)	133
D.A.	-	-	-	-	-	14 (10.5)	3 (2.3)	27 (20.3)	89 (66.9)	133
Total	-	-	-	-	-	880 (73.3)	25 (2.1)	141 (11.7)	154 (12.8)	1200
